# Comparison of weekly and daily recall of pain as an endpoint in a randomized phase 3 trial of cabozantinib for metastatic castration-resistant prostate cancer

**DOI:** 10.1177/17407745211009547

**Published:** 2021-04-22

**Authors:** Elisabeth M Schaffer, Ethan M Basch, Gisela M Schwab, Antonia V Bennett

**Affiliations:** 1 University of Colorado School of Medicine, Aurora, CO, USA; 2 Department of Medicine, University of North Carolina, Chapel Hill, NC, USA; 3 Exelixis Inc, Alameda, CA, USA; 4 Department of Health Policy and Management, University of North Carolina, Chapel Hill, NC, USA

**Keywords:** Mental recall, randomized phase 3 drug trial, cancer pain, patient-reported outcome

## Abstract

**Introduction:**

Scant evidence reveals whether the use of weekly versus daily pain ratings leads to meaningful differences when measuring pain as a clinical trial outcome. We compared the ability of weekly ratings and descriptors of daily ratings to evaluate pain as an endpoint in a randomized phase 3 drug trial.

**Methods:**

Participants (*n* = 119) with metastatic castration-resistant prostate cancer were randomized to treatment arms and rated their pain on the average and at its worst during a baseline week and at weeks 3, 6, and 12 of study treatment. For each reporting period, participants rated their pain daily for 7 days. On day 7, participants rated their pain over the prior 7 days. We estimated mean differences and intraclass correlation coefficients of the weekly ratings and the mean and the maximum daily ratings. We compared the ability of the weekly ratings and the daily rating descriptors to detect change in pain and evaluated the agreement of the weekly rating and the mean daily rating of pain at its worst to detect treatment response.

**Results:**

For both pain constructs, the weekly rating was consistently higher than the mean daily rating and lower than the maximum daily rating yet was moderately to highly correlated with both daily rating descriptors (intraclass correlation coefficient range = 0.55–0.94). The weekly rating and the daily rating descriptors consistently detected change in pain for the study sample and participant subgroups. Substantial agreement existed between the weekly rating and the mean daily rating of pain at its worst when used with trial protocol opioid criteria to detect treatment response (Cohen’s *κ* = 0.71).

**Conclusion:**

Use of daily over weekly ratings delivered no added benefit in evaluating pain in this clinical trial. This study is the first to compare weekly and daily recall to measure pain as an endpoint in a randomized phase 3 drug trial, and the pattern of differences in ratings that we observed is consistent with other recent evaluations of weekly and daily symptom reporting.

## Introduction

Pain is a common symptom of metastatic cancer that affects physical functioning and quality of life.^1–5^ Patients with advanced cancer are particularly susceptible to debilitating pain, and identifying agents to control and relieve pain for these patients is a critical research need.^3,5^ Although pain has often been included as a secondary endpoint in novel drug trials to support investigation of primary survival endpoints, some secondary endpoint analyses have been designed to lead to formal indications for pain palliation.^6–12^ Evidence that palliative benefits of novel agents are not always consistent with survival advantages has also promoted the rigorous investigation of pain in clinical trials, including as a primary endpoint in randomized phase 3 drug trials.^7,13–15^

The US Food and Drug Administration (FDA) encourages the investigation of pain in clinical trials and has outlined guidance for submitting patient-reported outcome (PRO) data as evidence of drug effectiveness.^16^ Because pain ratings come directly from patients, a recall period must be defined when designing trial protocols to evaluate pain as study endpoint. To avoid risks of recall bias, FDA guidance indicates that shorter recall periods are generally preferred. PRO theory provides general guidance for identification of appropriate recall periods, which depends largely on the context of use.^17^ The empirical literature investigating the impact of various recall periods on the assessment of pain in cancer clinical trials is small.

Prior research has detected differences in ratings of recalled pain and daily or momentary ratings of the same pain. Multiple studies have found that ratings of recalled average pain tend to be higher than the average of daily or momentary ratings.^18–26^ Fewer studies have investigated recall of pain at its worst. Results from those that have are mixed with evidence indicating that recall of pain at its worst could be higher^25,26^ or lower^27,28^ than maximum daily or momentary ratings (i.e. the expected mathematical equivalent). While the direction of the differences in ratings is useful to identify cognitive biases that influence recall, the magnitude of the differences in ratings and the correlation of ratings are also important to consider when appraising a recall period for measuring pain as a clinical outcome. Studies to date have largely found small differences and moderate to high correlation of ratings that are recalled over a period of 1 week or less when compared with daily or momentary ratings.^20,23–26^ Yet, scant evidence confirms whether the use of a short-term recall period versus daily or momentary assessment leads to differences when measuring treatment outcomes.

This study is the first to evaluate short-term recall to measure pain as an endpoint in a randomized phase 3 drug trial. The trial evaluated the safety and efficacy of cabozantinib versus mitoxantrone–prednisone to reduce pain from symptomatic metastatic castration-resistant prostate cancer.^14^ Participants provided weekly and daily ratings of their pain on the average and at its worst during multiple 7-day reporting periods throughout the trial. The trial thus provided a rare opportunity to compare weekly and daily ratings of two pain constructs and investigate whether differences in ratings translated to meaningful differences in pain assessment.

This article presents results from four comparative analyses: (1) comparison of weekly ratings with summary descriptors (i.e. the mean and the maximum) of daily pain ratings; (2) comparison of the weekly ratings and the daily rating descriptors to detect change in pain for the sample; (3) comparison of the weekly ratings and the daily rating descriptors to detect change in pain within and across treatment subgroups; and (4) comparison of the weekly rating and mean daily rating of pain at its worst to detect treatment response. In Analysis 1, we tested two hypotheses based on the mathematical expected value of the weekly rating for each pain construct. For pain on the average, we hypothesized that the weekly rating would be more similar to the mean daily rating. For pain at its worst, we hypothesized that the weekly rating would be more similar to the maximum daily rating.

## Methods

### Setting and participants

The trial (COMET-2; NCT01522443) enrolled participants from health centers in the United States, the United Kingdom, Ireland, Australia, and Canada. Adult (≥18 years) patients were eligible to participate if they had a pathological diagnosis of metastatic castration-resistant prostate cancer, serum testosterone levels <50 ng/dL, history of progression after prior treatment with docetaxel and either abiraterone or enzalutamide, and pain requiring opioid narcotic intervention. Patients were ineligible to participate if they received prior treatment with cabozantinib or mitoxantrone. Patients who met eligibility criteria and provided written informed consent to participate were randomized 1:1 to receive cabozantinib or mitoxantrone–prednisone.

Trial enrollment began in March 2012 and was terminated in October 2014 due to failure of a companion trial to observe a significant overall survival benefit of cabozantinib relative to prednisone.^29^ An ethics committee at each enrollment site approved the trial protocol, and the trial was conducted in accordance with Good Clinical Practice Guidelines and the Declaration of Helsinki.

### Data collection

Participants rated their pain during a 7-day baseline period prior to randomization and during 7-day reporting periods in weeks 3, 6, and 12 of study treatment. For each day of the reporting periods, participants rated their pain over the past 24 h according to two constructs—pain on the average and pain at its worst. Participants rated their pain using an 11-point numerical rating scale that ranged from 0 to 10, with 0 representing “No Pain” and 10 representing “Pain as Bad as You Can Imagine.” The survey items came from the Brief Pain Inventory (BPI) Short Form, an instrument that is widely used in contemporary pain studies and whose development and validation have been well-documented.^30–32^ On the last day of each reporting period, participants rated their pain over the past 7 days. The weekly items exactly mirrored the daily items except that the recall period was the “past 7 days” rather than the “past 24 hours” (Table 1).

**Table 1.** Daily and weekly survey items, listed by construct.

Survey item	Item wording
Pain on the average	
BPI daily item	Please rate your pain on the AVERAGE in the last 24 h, using a scale from 0 to 10, where 0 meansno pain and 10 means pain as bad as you can imagine.
Adapted forweekly report	Please rate your pain on the AVERAGE in the last 7 days, using a scale from 0 to 10, where 0 meansno pain and 10 means pain as bad as you can imagine.
Pain at its worst	
BPI daily item	Please rate your pain at its WORST in the last 24 h, using a scale from 0 to 10, where 0 means nopain and 10 means pain as bad as you can imagine.
Adapted forweekly report	Please rate your pain at its WORST in the last 7 days, using a scale from 0 to 10, where 0 meansno pain and 10 means pain as bad as you can imagine.

BPI: Brief Pain Inventory.

Pain was assessed via a telephone survey that was automated with an interactive voice response system. The interactive voice response system was chosen for capturing data because it was easily accessible, allowed participants to rate their pain independently, and has achieved high levels of compliance.^33–35^

### Statistical analyses

Analyses were limited to weeks of reporting that included the weekly rating and at least four daily pain ratings. Weeks of reporting that were missing a weekly rating or that had fewer than four daily ratings were excluded from analysis, regardless of the number of other (i.e. daily or weekly) ratings that were provided that week. We defined statistical significance as *p* < 0.05 with two-sided 95% confidence intervals (CIs) and estimated robust standard errors that were clustered at the individual level to account for correlation from multiple ratings per patient.

#### Comparison of the weekly ratings with descriptors of the daily ratings

For each construct, we compared the weekly rating with the mean of the daily ratings and the maximum of the daily ratings (i.e. the most severe rating) in the corresponding 7-day reporting period. The mean daily rating is often used to score weeklong symptom severity in PRO analyses, whereas the maximum daily rating is often used to analyze adverse events. Absent of cognitive bias, the weekly rating of pain on the average is expected to approximate the mean daily rating of pain on the average, and the expected value of the weekly rating of pain at its worst is the maximum daily rating of pain at its worst.

To determine whether systematic differences in the weekly rating and each daily rating descriptor were detectable in our data, we estimated linear regression models that analyzed variation in the ratings by rating type, that is, weekly or daily. The coefficient for the rating type variable indicated the mean difference in the weekly rating relative to the daily rating descriptor, and the *t*-statistic associated with the coefficient tested the hypothesis of no difference in the weekly rating and the daily rating descriptor. Because statistical significance depends on sample size and differences that are not clinically meaningful can be statistically significant, we estimated Cohen’s *d* statistics to evaluate the effect sizes of the differences. Cohen’s *d* standardizes the mean difference in ratings using pooled standard deviations, and effect sizes are interpreted according to the absolute value of the *d*-statistic as follows: 0 to <0.2 indicates a trivial effect, 0.2 to <0.5 indicates a small effect, 0.5 to <0.8 indicates a moderate effect, and ≥0.8 indicates a large effect.^36^

We assessed strength of association between the weekly rating and each descriptor of daily ratings, that is, the mean value and the maximum value, by estimating intraclass correlation coefficients (ICCs). The ICC takes into account multiple ratings of the same phenomenon by a single rater. We estimated ICCs for the weekly rating and each daily rating descriptor per reporting period. We determined that an ICC indicated high agreement if it and its corresponding 95% CI were greater than 0.70, in accordance with standard practice. We also compared the 95% CIs of the ICCs for each rating pair to assess whether the correlation of the weekly rating and the mean daily rating was significantly greater or less than that of the weekly rating and the maximum daily rating, as could be determined by no overlap in the CIs of the ICCs for each rating pair.

#### Comparison of the weekly ratings and the daily rating descriptors to detect change in pain for the sample

To assess the ability of the weekly rating and the daily rating descriptors to detect change in pain, we estimated mean differences in response-scale points from baseline to week 12 of study treatment. We estimated linear regression models that analyzed variation in ratings by time period. The coefficient for the independent time period variable indicated the mean difference in the value of each rating at week 12 relative to baseline, and the *t*-statistic associated with the coefficient tested against the hypothesis of no difference in rating values over time. We evaluated the effect size of the change in pain by estimating Cohen’s *d*-statistic. To evaluate the robustness of our results to reporting differences over the two periods, we conducted a sensitivity analysis that included only observations from participants who provided evaluable ratings at both baseline and week 12.

#### Comparison of the weekly ratings and the daily rating descriptors to detect change in pain within and across treatment subgroups

We compared the ability of the weekly rating and the daily rating descriptors to detect change in pain within treatment subgroups by stratifying the ratings by participants’ randomized treatment assignments. We then estimated change in pain within each treatment subgroup from baseline to week 12. To compare the ability of the ratings to detect differences in change in pain across treatment subgroups, we estimated linear regression models that analyzed variation in ratings by reporting period and by randomized treatment assignment and that included an interaction term between the reporting period and the randomized treatment assignment. The *t*-statistic of the interaction term coefficient tested against the hypothesis of no difference across treatment subgroups in the change in pain from baseline to week 12.

#### Comparison of the weekly rating and mean daily rating of pain at its worst to detect treatment response

The trial protocol defined treatment response as a ≥30% reduction in pain from baseline at week 6 that was confirmed at week 12, without increased opioid use. Pain at each reporting period was defined as the mean daily rating of pain at its worst. Therefore, in this analysis, we compared the proportion of participants with a treatment response using a definition based on the mean daily rating of pain at its worst with the proportion of participants with a treatment response using a definition based on the weekly rating of pain at its worst. We estimated Cohen’s kappa (*κ*) coefficient to assess the agreement of the definitions to identify treatment responders. We interpreted the value of the *κ*-statistic according to Landis and Koch’s (1977) guidelines by which: < 0.20 indicates slight agreement, 0.21–0.40 indicates fair agreement, 0.41–0.60 indicates moderate agreement, 0.61–0.80 indicates substantial agreement, and 0.81–1.00 indicates almost perfect agreement.^37^ We first conducted the analysis taking into account participant opioid use, in accordance with the trial protocol definition of treatment response. We then repeated the analysis excluding information about participant opioid use to allow for comparison of the ratings alone to detect pain reduction.

## Results

### Patient characteristics

In total, 119 participants were enrolled in the randomized trial (Table 2). The median age was 65 years (range = 44–80), and most participants were White (84%) and non-Hispanic (96%). Participants were evenly divided between those whose mean daily rating of pain at its worst ranged from 4 to 6 (50%) and from >6 to 8 (50%). The majority of participants (65%) rated their baseline overall health-related quality of life from 4 to 6 on a 0–10 scale. Sixty-one participants (51%) were randomized to cabozantinib and 58 (49%) were randomized to mitoxantrone–prednisone.

**Table 2.** Participant demographic and baseline health characteristics.

	*N* (%)
Total participants	119
Age group (years)	
44–64	50 (42)
65–75	58 (49)
>75	11 (9)
Country of enrollment	
United States	70 (59)
United Kingdom/Ireland	21 (18)
Australia	18 (15)
Canada	10 (8)
Race	
White	100 (84)
Black or African American	11 (9)
Asian	4 (3)
American Indian or Alaska Native	1 (1)
Multiple	1 (1)
Other/not reported	2 (2)
Ethnicity	
Not Hispanic or Latino	114 (96)
Hispanic or Latino	2 (2)
Unknown/not reported	3 (3)
Eastern Cooperative OncologyGroup Performance Status	
0: no functional limitations	19 (16)
1: unable to perform strenuous activities	82 (70)
2: unable to work or performstrenuous activities	16 (14)
Self-rated pain at its worst (BPI ShortForm item 3), mean rating of 7 days^a^	
4–5	25 (21)
>5–6	35 (29)
>6–7	33 (28)
>7–8	26 (22)
Self-rated overall health-related qualityof life (LASA), rating on a 0–10 scale	
0–3	11 (10)
4–6	74 (65)
7–10	29 (25)

BPI: Brief Pain Inventory; LASA: linear analog self-assessment.

a Patients who provided at least four daily ratings over a 7-day screening period and whose mean daily rating of pain at its worst was at least four and no more than eight were eligible to participate.

### Data characteristics

Participants provided 431 weeks of pain data from baseline through week 12 of study treatment. Thirty-one (7%) weeks were missing the weekly rating and were excluded from analysis. One week that included the weekly rating had fewer than four daily ratings for pain on the average and was excluded from analysis of that construct. The 400 weeks included in the analysis for at least one pain construct comprised 199 (50%) weeks of pain reports from participants randomized to mitoxantrone–prednisone and 201 (50%) weeks of pain reports from participants randomized to cabozantinib. All trial participants provided at least 1 week of evaluable data. Attrition due to limited treatment efficacy, adverse events, and other causes contributed to fewer weeks of data over time yet completion of ratings by enrolled participants was high over all reporting periods; 107 participants (93%) provided weekly and at least four daily ratings at week 3, 100 (93%) at week 6, and 80 (89%) at week 12.

Data included in the analysis comprised 312 (78%) weeks in which seven daily ratings were provided and 88 (22%) weeks in which four, five, or six daily ratings were provided. No significant differences were observed when comparing differences in the weekly rating and the mean or the maximum daily rating for weeks with no missing daily ratings and weeks missing one, two, or three daily ratings. The within-person variation per week of daily ratings, calculated as the mean standard deviation of the daily ratings provided per person-week of analysis, was 0.60 response-scale points for pain on the average and was 0.85 response-scale points for pain at its worst. Table 3 presents the sample mean, standard deviation, median, and interquartile range of the weekly rating and of the daily rating descriptors for each construct across and for each reporting period through week 12 of study treatment.

**Table 3.** Characteristics of pain ratings across reporting periods and at baseline, week 6, and week 12 of study treatment.

	Aggregate^a^	Baseline	Week 6	Week 12
Pain on the average, mean (SD)				
Weekly rating	3.60 (1.82)	4.57 (1.48)	3.17 (1.85)	2.59 (1.79)
Mean of 7 days	3.40 (1.71)	4.39 (1.24)	2.89 (1.68)	2.43 (1.68)
Maximum of 7 days	4.24 (2.00)	5.31 (1.52)	3.70 (1.95)	3.13 (2.01)
Pain on the average, median (IQR)				
Weekly rating	4 (2–5)	5 (4–5)	3 (2–4)	3 (1–4)
Mean of 7 days	3.57 (2.15–4.43)	4.29 (3.71–5.14)	2.77 (1.86–3.86)	2.29 (1.00–3.51)
Maximum of 7 days	4 (3–6)	5 (4–7)	3 (3–5)	3 (2–4)
Pain at its worst, mean (SD)				
Weekly rating	5.58 (2.24)	6.67 (1.64)	5.01 (2.27)	4.49 (2.52)
Mean of 7 days	4.81 (1.97)	6.00 (1.11)	4.19 (2.03)	3.70 (2.11)
Maximum of 7 days	5.96 (2.16)	7.11 (1.23)	5.39 (2.32)	4.78 (2.48)
Pain at its worst, median (IQR)				
Weekly rating	6 (4–7)	7 (6–8)	5 (3–6)	4.5 (3–7)
Mean of 7 days	5.00 (3.46–6.29)	6.00 (5.14–6.86)	4.21 (2.69–5.57)	3.33 (2.29–5.43)
Maximum of 7 days	6 (5–8)	7 (6–8)	6 (4–7)	5 (3–7)

SD: standard deviation; IQR: interquartile range.

a Includes ratings provided at baseline, which is prior to randomization, and at weeks 3, 6, and 12 of study treatment.

### Rating comparisons

#### Comparison of the weekly ratings with descriptors of the daily ratings

**Figure 1.**
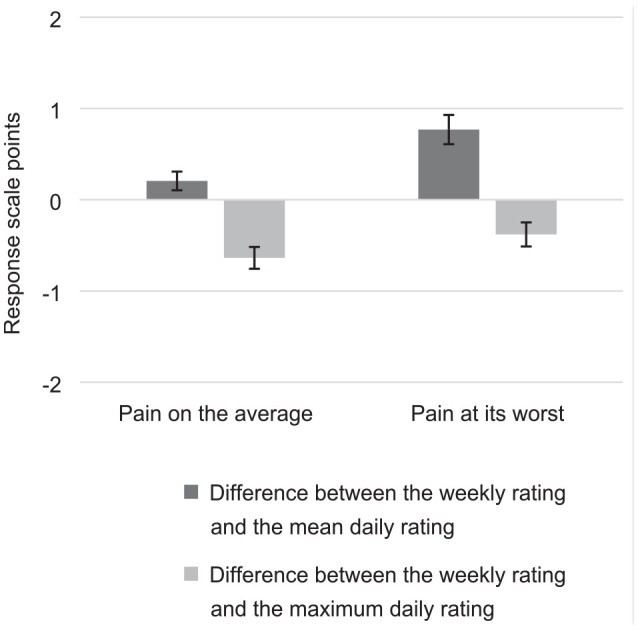
Mean differences between the weekly rating and descriptors of the daily ratings.

Analyzing pain ratings in aggregate from baseline through week 12 of study treatment, we found that the mean difference (standard error (SE)) between the weekly and mean daily ratings was 0.21 (0.05) response-scale points for pain on the average and 0.77 (0.08) response-scale points for pain at its worst (Figure 1). The mean difference between the weekly and maximum daily ratings was −0.64 (0.06) response-scale points for pain on the average and −0.38 (0.07) response-scale points for pain at its worst. The effect size of the mean difference between the weekly and mean daily rating was 0.12 for pain on the average and 0.36 for pain at its worst. The effect size of the mean difference between the weekly and maximum daily rating was −0.33 for pain on the average and −0.17 for pain at its worst.

We observed similar nonzero differences between the weekly rating and the daily rating descriptors (all *p* < 0.05) when analyzing pain report data by each 7-day reporting period (Supplementary Figures S1 and S2). ICCs indicated moderate to high correlation of the weekly rating and the daily rating descriptors (Table 4). For pain on the average, the ICCs were consistently higher when comparing the weekly rating with the mean daily rating rather than the maximum daily rating. For pain at its worst, the ICCs were consistently higher when comparing the weekly rating with the maximum daily rating rather than the mean daily rating. Yet, the 95% CIs of the ICCs for the weekly rating and each descriptor of daily ratings overlapped considerably, and we could not conclude that the weekly rating was more strongly correlated with the mean or the maximum daily rating for either pain construct.

**Table 4.** Differences and correlation of weekly ratings and descriptors of daily ratings, by reporting period.

	Weekly rating vs mean of 7 days			Weekly rating vs maximum of 7 days		
Construct	Mean difference	Effect size^a^	ICC (95% CI)	Mean difference	Effect size^a^	ICC (95% CI)
Pain on the average						
Baseline	0.18	0.13	0.77 (0.68–0.83)	−0.74	−0.49	0.73 (0.63–0.80)
Week 3	0.19	0.12	0.85 (0.78–0.89)	−0.71	−0.40	0.83 (0.76–0.88)
Week 6	0.28	0.16	0.89 (0.84–0.93)	−0.53	−0.28	0.83 (0.75–0.88)
Week 12	0.17	0.10	0.93 (0.90–0.96)	−0.52	−0.27	0.91 (0.87–0.94)
Pain at its worst						
Baseline	0.68	0.49	0.55 (0.41–0.67)	−0.43	−0.30	0.69 (0.57–0.77)
Week 3	0.80	0.42	0.80 (0.72–0.86)	−0.39	−0.20	0.86 (0.81–0.90)
Week 6	0.82	0.38	0.77 (0.68–0.84)	−0.38	−0.17	0.79 (0.70–0.85)
Week 12	0.79	0.34	0.87 (0.80–0.92)	−0.29	−0.11	0.94 (0.92–0.96)

ICC: intraclass correlation coefficient; CI: confidence interval.

a Calculated as Cohen’s *d*.

#### Comparison of the weekly ratings and the daily rating descriptors to detect change in pain for the sample

All rating types detected a significant response-scale point reduction in pain from baseline to week 12 of study treatment (Table 5). The effect sizes for the change for both pain constructs were large across all ratings. The absolute value of the *t*-statistics and effect sizes were the largest for the mean daily rating yet the significance was the same across all ratings. These findings held when ratings were only included from participants who provided evaluable ratings at both baseline and week 12 (Table S1).

**Table 5.** Change in pain from baseline to week 12 of treatment, by rating type.

Construct	Mean change in ratings (SE)	*t*-statistic (significance)	Effect size^a^
Pain on the average			
Weekly rating	−1.98 (0.22)	−9.05 (<0.001)	−1.22
Mean of 7 days	−1.97 (0.19)	**−10.18 (<0.001)**	**−1.37**
Maximum of 7 days	−2.21 (0.24)	−9.32 (<0.001)	−1.27
Pain at its worst			
Weekly rating	−2.19 (0.31)	−7.03 (<0.001)	−1.06
Mean of 7 days	−2.30 (0.24)	**−9.66 (<0.001)**	**−1.44**
Maximum of 7 days	−2.33 (0.29)	−8.14 (<0.001)	−1.26

SE: standard error.

a Calculated as Cohen’s *d. t*-statistics and effect sizes with the largest absolute value are in bold.

#### Comparison of the weekly ratings and the daily rating descriptors to detect change in pain within and across subgroups

All rating types detected significant response-scale point reductions in pain from baseline to week 12 that corresponded to large effect sizes when stratifying ratings by randomized treatment assignment (Table S2). The effect sizes of the change in pain within subgroups were the largest for the mean daily rating. No rating types detected significant differences in change in pain across randomized treatment arms (all *p* > 0.05).

#### Comparison of the weekly rating and the mean daily rating to detect treatment response

21% of participants (*n* = 17) were identified as treatment responders using the trial protocol definition of pain response based on the mean daily rating of pain at its worst and that required no increases in participant opioid use. Taking the same opioid criteria into account, 15% of participants (*n* = 12) were identified as treatment responders using the weekly rating of pain at its worst. Cohen’s *κ*-statistic assessing the agreement of the approaches to detect treatment response was 0.71 (*p* < 0.01), indicating substantial agreement. When the trial protocol criteria regarding opioid use were not taken into account, 38% of participants (*n* = 31) were identified who experienced a ≥30% pain reduction using the mean daily rating of pain at its worst and 28% of participants (*n* = 23) were identified who experienced a ≥30% pain reduction using the weekly rating of pain at its worst. Cohen’s *κ*-statistic assessing the agreement of the ratings to detect pain reduction was 0.56 (*p* < 0.01), indicating moderate agreement.

## Discussion

For both pain constructs, the weekly rating was consistently higher than the mean daily rating and lower than the maximum daily rating. While the differences were significant, the effect sizes of the differences ranged from trivial to small, and the correlation of the weekly rating and the daily rating descriptors was mostly high. The effect sizes of the differences in ratings and the magnitude of the ICCs for the rating pairs suggested that the weekly rating was more similar to the mean daily rating for pain on the average and to the maximum daily rating for pain at its worst, yet we could not conclude that the weekly rating was more strongly correlated with the mean or the maximum daily rating for either construct given overlap of the 95% CIs of the ICCs for the rating pairs.

The pattern of differences in ratings that we observed for pain on the average is consistent with other evaluations of pain recall.^20,21,24,26,28^ For pain at its worst, prior findings have been mixed regarding the direction of the differences in ratings of recalled pain compared to maximum daily or momentary ratings. Our findings are, however, consistent with several studies, including a growing number of studies investigating symptom recall more broadly.^27,28,38–40^ Findings that ratings of recalled average pain tend to be higher than the mean of daily or momentary ratings of average pain have often been attributed to a cognitive bias that recall is unduly influenced by episodes of peak pain.^18,19,24,41^ For recall of pain at its worst (i.e. recall of peak pain), the field has largely been silent regarding cognitive biases. Based on our findings and those of a number of prior studies, we think that recall of pain at its worst is subject to a slight averaging bias.

Our results could be limited if participants’ provision of daily ratings influenced their weekly ratings. Three symptom recall studies have investigated the possibility of weekly rating reactivity by randomizing participants to groups that did and did not provide daily or momentary ratings with weekly ratings.^39,42,43^ In these studies, the weekly ratings did not differ significantly between groups, and we think little risk of weekly rating reactivity exists in this study.

The opportunities to compare the weekly rating and the daily rating descriptors to assess pain in the clinical trial were unique study strengths. All rating types detected a mean pain reduction from baseline to week 12 that did not differ significantly across treatment arms. Using the trial protocol opioid criteria alongside pain ratings, we found substantial agreement of definitions of treatment response that were based on the weekly and mean daily ratings of pain at its worst. Excluding opioid criteria, more participants were identified who experienced pain reduction, and the agreement of the weekly and mean daily ratings to detect pain reduction was moderate. In both cases, fewer participants met the ≥30% pain reduction threshold using the weekly rating, suggesting that it may detect pain reduction more conservatively. Given the controlled nature of randomized experiments, it is unlikely that variability in ratings that was introduced by excluding opioid criteria would be unaccounted for in future trials, yet greater variability in ratings could be more common in clinical practice.

One study limitation was underrepresentation of racial and ethnic minorities. Gender and age-based demographic variation has previously been detected in the magnitude of the differences between symptom ratings recalled over a period of 1 week or longer and daily ratings.^38,44,45^ The sign of the differences at least remained consistent with the broader population. Greater representation of minority groups would enable further investigation of demographic variation in ratings and strengthen future studies.

## Conclusion

This study confirmed that weekly ratings and daily rating descriptors of pain intensity differ. Yet, the differences were minor and consistent across reporting periods such that the ratings led to consistent results regarding change in pain and treatment response. Although daily symptom assessment is increasingly feasible due to electronic symptom reporting technologies and is warranted when measuring variation of ratings within a week, we did not discover added benefits of daily relative to weekly ratings to evaluate participant pain in this clinical trial.

## Supplemental Material

sj-pdf-1-ctj-10.1177_17407745211009547 – Supplemental material for Comparison of weekly and daily recall of pain as an endpoint in a randomized phase 3 trial of cabozantinib for metastatic castration-resistant prostate cancer Click here for additional data file. Supplemental material, sj-pdf-1-ctj-10.1177_17407745211009547 for Comparison of weekly and daily recall of pain as an endpoint in a randomized phase 3 trial of cabozantinib for metastatic castration-resistant prostate cancer by Elisabeth M Schaffer, Ethan M Basch, Gisela M Schwab and Antonia V Bennett in Clinical Trials
