# Supplementary material for: Comparison of weekly and daily recall of pain as an endpoint in a randomized phase 3 trial of cabozantinib for metastatic castration-resistant prostate cancer
Source: Clin Trials. 2021 Apr 22;18(4):408–16. doi: 10.1177/17407745211009547 (PMC8290982; doi:10.1177/17407745211009547)
Supplement: sj-pdf-1-ctj-10.1177_17407745211009547 – Supplemental material for Comparison of weekly and daily recall of pain as an endpoint in a randomized phase 3 trial of cabozantinib for metastatic castration-resistant prostate cancer [file sj-pdf-1-ctj-10.1177_17407745211009547.pdf]

Figure S1. Weekly ratings of pain on the average relative to the mean and maximum daily ratings for each 7-day reporting interval.

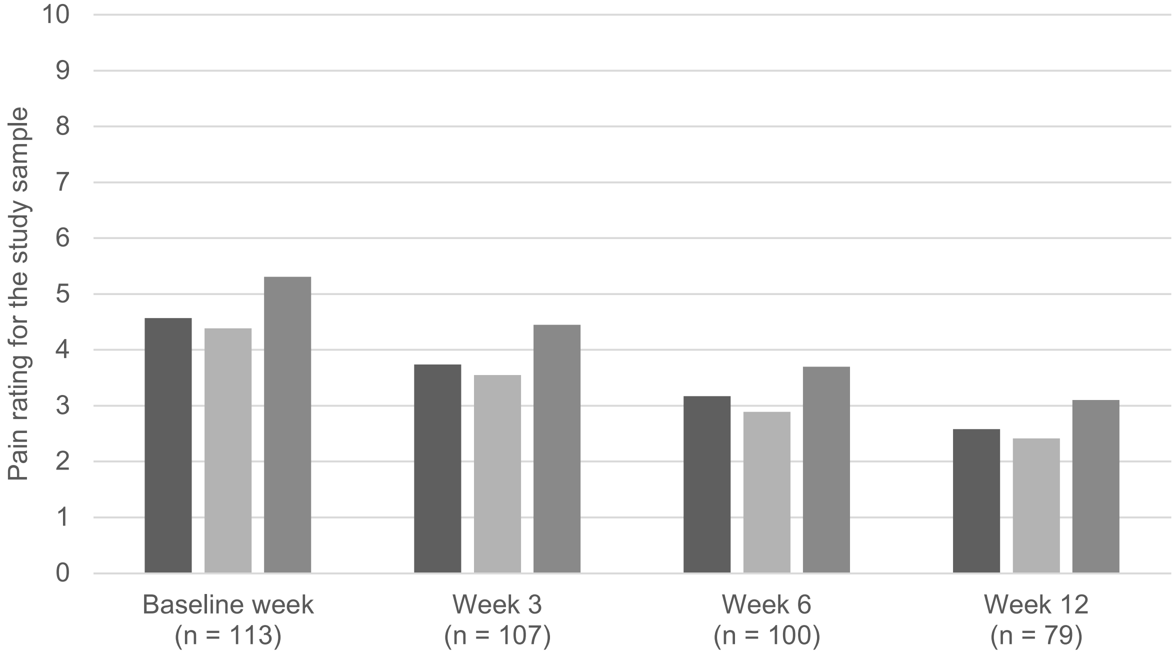

Figure legend

■ Weekly rating ■ Mean of 7 days ■ Maximum of 7 days

Figure S2. Weekly ratings of pain at its worst relative to the mean and maximum daily ratings for each 7-day reporting interval.

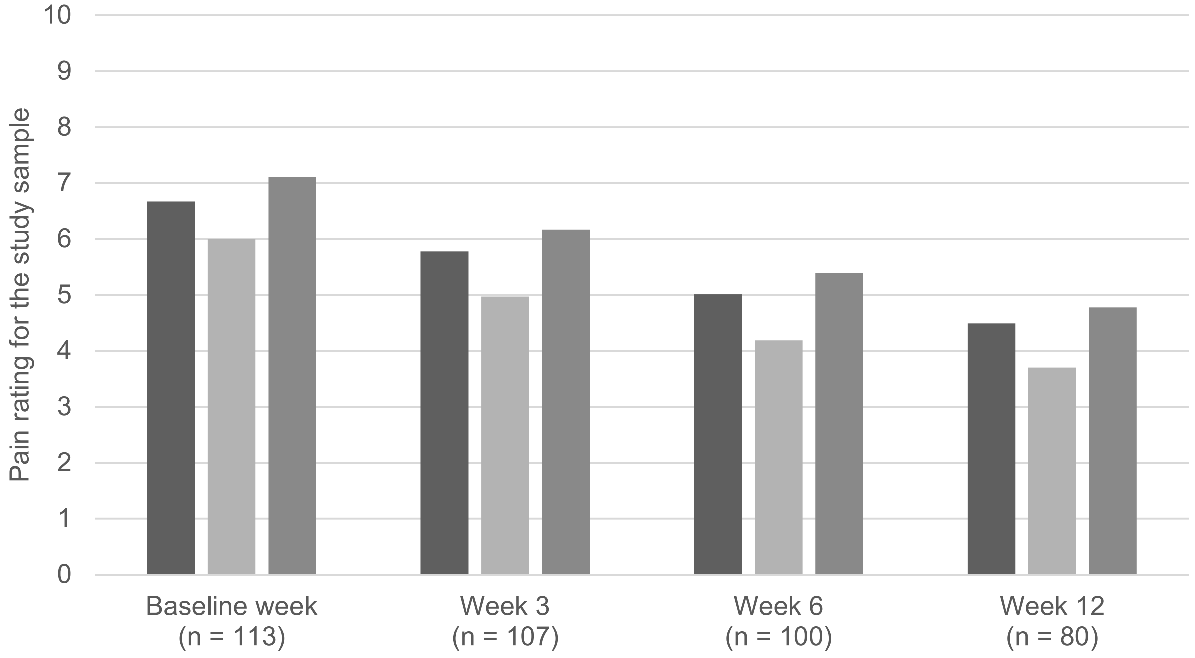

Figure legend

■ Weekly rating ■ Mean of 7 days ■ Maximum of 7 days

Table S1. Change in pain from baseline to week 12, by rating type and including only observations from participants who provided evaluable ratings at both time points.

**Construct**	Change in rating Mean (SE)	*T*-statistic (Significance)	Effect size[a]
**Pain on the average**			
Weekly rating	-1.86 (0.22)	-8.33 (<0.001)	-1.16
Mean of 7 days	-1.91 (0.19)	**-9.79 (<0.001)**	**-1.32**
Maximum of 7 days	-2.09 (0.23)	-9.10 (<0.001)	-1.24
**Pain at its worst**			
Weekly rating	-2.21 (0.33)	-6.61 (<0.001)	-1.03
Mean of 7 days	-2.34 (0.24)	**-9.71 (<0.001)**	**-1.41**
Maximum of 7 days	-2.43 (0.29)	-8.34 (<0.001)	-1.27

SE: standard error.

[a] Calculated as Cohen's *d*. *T*-statistics and effect sizes with the largest absolute value are in bold.

Table S2. Change in pain from baseline to week 12 of treatment, by rating type and stratified by randomized treatment assignment.

	Randomized to cabozantinib			Randomized to mitoxantrone-prednisone		
**Construct**	Change in rating Mean (SE)	*T*-statistic (Significance)	Effect size[a]	Change in rating Mean (SE)	*T*-statistic (Significance)	Effect size[a]
**Pain on the average**						
Weekly rating	-2.18 (0.29)	-7.46 (<0.001)	-1.34	-1.78 (0.33)	-5.40 (<0.001)	-1.09
Mean of 7 days	-2.13 (0.26)	**-8.31 (<0.001)**	**-1.50**	-1.81 (0.29)	**-6.18 (<0.001)**	**-1.23**
Maximum of 7 days	-2.37 (0.33)	-7.25 (<0.001)	-1.35	-2.04 (0.35)	-5.88 (<0.001)	-1.17
**Pain at its worst**						
Weekly rating	-2.46 (0.43)	-5.76 (<0.001)	-1.26	-1.91 (0.45)	-4.21(<0.001)	-0.89
Mean of 7 days	-2.62 (0.32)	**-8.06 (<0.001)**	**-1.76**	-1.98 (0.35)	**-5.70 (<0.001)**	**-1.17**
Maximum of 7 days	-2.71 (0.40)	-6.73 (<0.001)	-1.51	-1.94 (0.41)	-4.79 (<0.001)	-1.02

SE: standard error.

[a] Calculated as Cohen's *d*. *T*-statistics and effect sizes with the largest absolute value are in bold.

Table S3. Change in pain from baseline to week 12 of treatment, by rating type and stratified by baseline pain ratings.

	Provided a baseline pain rating ≤6			Provided a baseline pain rating >6		
**Construct**	Change in rating Mean (SE)	*T*-statistic (Significance)	Effect size[a]	Change in rating Mean (SE)	*T*-statistic (Significance)	Effect size[a]
**Pain on the average**						
Weekly rating	-1.88 (0.22)	-8.69 (<0.001)	1.32	-3.27 (0.97)	-3.37 (=0.007)	2.48
Mean of 7 days	-1.80 (0.19)	**-9.56 (<0.001)**	**1.44**	-3.29 (0.88)	-3.74 (=0.002)	2.19
Maximum of 7 days	-1.88 (0.24)	-7.77 (<0.001)	1.34	-3.21 (0.54)	**-6.00 (<0.001)**	**2.56**
**Pain at its worst**						
Weekly rating	-1.09 (0.38)	-2.86 (=0.006)	0.70	-3.20 (0.43)	-7.46 (<0.001)	1.75
Mean of 7 days	-1.74 (0.27)	**-6.39 (<0.001)**	**1.44**	-3.01 (0.39)	**-**7.67 (<0.001)	**1.97**
Maximum of 7 days	-1.28 (0.34)	-3.79 (=0.001)	1.06	-3.00 (0.38)	**-7.89 (<0.001)**	1.69

SE: standard error.

[a] Calculated as Cohen's *d*. *T*-statistics and effect sizes with the largest absolute value are in bold.
